# Tailoring carbon shell thickness in graphene–Li_2_S–carbon nanocomposite cathodes for enhanced polysulfide control and electrochemical stability

**DOI:** 10.1039/d5ra07689g

**Published:** 2026-01-09

**Authors:** Farag M. A. Altalbawy, I. B. Sapaev, Fadel F. Saied, Paul Rodrigues, Rekha M. M., Laxmidhar Maharana, P. Grace Kanmani Prince, Gaganjot Kaur, Malik Bader Alazzam, Shayan Amiri

**Affiliations:** a Renewable Energy and Environmental Technology Center, University of Tabuk Tabuk Saudi Arabia; b Head of the Department of Physics and Chemistry, Tashkent Institute of Irrigation and Agricultural Mechanization Engineers, National Research University Tashkent Uzbekistan; c Scientific Researcher of the University of Tashkent for Applied Science Uzbekistan; d School of Engineering, Central Asian University Tashkent 111221 Uzbekistan; e Western Caspian University, Scientific Researcher Baku Azerbaijan; f Department of Chemistry, College of Chemistry, The Islamic University Najaf Iraq; g Department of Computer Engineering, College of Computer Science, King Khalid University Al-Faraa Kingdom of Saudi Arabia; h Department of Chemistry and Biochemistry, School of Sciences, JAIN (Deemed to Be University) Bangalore Karnataka India; i Department of Pharmaceutical Sciences, Siksha ‘O’ Anusandhan (Deemed to Be University) Bhubaneswar Odisha-751030 India; j Department of Biomedical, Sathyabama Institute of Science and Technology Chennai Tamil Nadu India; k Department of Electronics and Communication Engineering, Chandigarh University Mohali Punjab India; l Faculty of Information Technology, Jadara University Irbid Jordan; m Young Researchers and Elite Club, Tehran University Tehran Iran sh.amiriacademic@gmail.com; n Sharda School of Engineering and Science, Sharda University Greater Noida UP India

## Abstract

Lithium–sulfur (Li–S) batteries are promising next-generation energy storage systems due to their high theoretical energy density and the abundance of sulfur; however, their practical application is severely limited by the poor electrical and ionic conductivity of Li_2_S and the dissolution of intermediate polysulfides. In this work, a comprehensive multiphysics simulation study is conducted to investigate the influence of carbon shell thickness (0–20 nm) on the electrochemical, thermal, and ionic performance of graphene–Li_2_S–carbon nanocomposite cathodes under experimentally realizable conditions (1C discharge rate and 35 °C). The model, developed using COMSOL Multiphysics, couples heat transfer, ion transport, and electric current conservation to capture the complex interactions governing cathode behavior. To ensure experimental relevance and reliability, the simulation results are rigorously validated against reported experimental voltage–capacity data for graphene–Li_2_S–carbon cathodes, achieving a low root mean square error of 0.09 V. The results reveal that a carbon shell thickness of approximately 10 nm provides an optimal balance between polysulfide confinement and lithium-ion transport, leading to minimized temperature rise, reduced ionic resistance, and improved current-density uniformity. By establishing a quantitative agreement with experimental literature, this study offers a predictive and experimentally grounded framework for the rational design and optimization of high-performance Li–S battery cathodes.

## Abstract 2

Multiphysics modeling reveals that carbon shell thickness controls ion transport, heat generation, and polysulfide confinement, with an optimal ∼10 nm shell enhancing Li–S battery performance.

## Graphical abstract

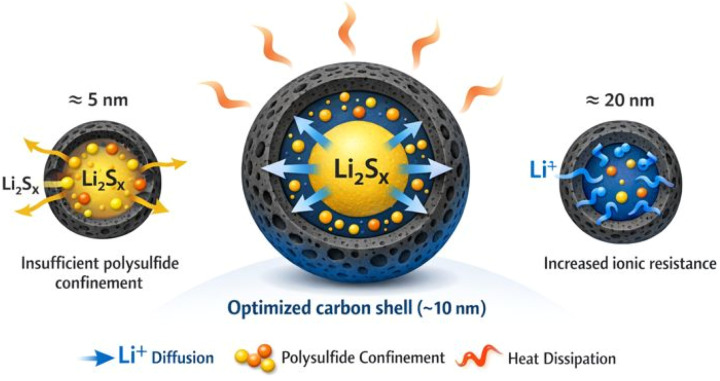

## 1. Introduction

Lithium–sulfur (Li–S) batteries are a promising energy storage technology due to their high theoretical energy density of 2600 Wh kg^−1^, far surpassing conventional lithium-ion batteries, and the use of sulfur, which is abundant, low-cost, and environmentally friendly.^1,2^ These attributes make Li–S batteries attractive for applications like electric vehicles and grid storage. However, their practical deployment is challenged by several limitations stemming from the complex electrochemical and chemical interactions at the cathode–electrolyte interface. The primary issues include the low electrical and ionic conductivity of sulfur (∼10^−5^ S m^−1^) and its discharge product, lithium sulfide (Li_2_S, ∼10^−8^ S m^−1^), which hinders efficient electron and ion transport. Additionally, the dissolution of intermediate polysulfides (*e.g.*, Li_2_S_8_, Li_2_S_6_) into the electrolyte causes the “shuttle effect,” where these species migrate to the anode, leading to active material loss, parasitic reactions, and electrolyte decomposition. These factors collectively degrade capacity retention and cycle life, limiting the battery's performance and longevity.^3–8^ To overcome these challenges, innovative cathode designs are essential to enhance conductivity, suppress polysulfide shuttling, and stabilize the electrode–electrolyte interface.^9–11^

Recent research has focused on composite cathodes incorporating carbon-based materials like graphene, carbon nanotubes, and porous carbon shells to address these issues.^12–14^ Graphene, with its high electrical conductivity (∼10^4^ S m^−1^) and tunable surface chemistry, is particularly effective, as it can physically or chemically trap polysulfides, reducing their dissolution into the electrolyte.^15–17^ Encapsulating Li_2_S particles with a carbon shell further improves electron and ion transport while acting as a protective barrier against electrolyte interactions, minimizing side reactions.^18–20^ However, the thickness of the carbon shell is a critical parameter. Thicker shells may increase tortuosity and resistance, impeding ion diffusion, while thinner shells might not sufficiently confine polysulfides, allowing leakage and shuttle effects.^21–23^ Optimizing this balance requires a deep understanding of the interplay between thermal, ionic, and electrical properties, which govern the cathode's electrochemical kinetics and chemical stability.^24–26^ Advanced multiphysics modeling, such as that enabled by COMSOL Multiphysics, can elucidate these interactions by simulating coupled phenomena like heat dissipation, ion transport, and electrochemical reactions, providing a predictive framework for designing high-performance Li–S cathodes.^27–29^ Such approaches are crucial for translating the theoretical potential of Li–S batteries into practical, high-efficiency energy storage systems.

Recent advances in lithium–sulfur battery research have highlighted that the electrochemical performance of composite sulfur cathodes is strongly governed by the structural characteristics of the carbon host and substrate. In particular, parameters such as carbon thickness, porosity, electrical conductivity, and host morphology critically influence lithium-ion transport, electronic percolation, and polysulfide confinement, especially under practical conditions such as high sulfur loading and lean electrolyte operation.^3,30^ Recent experimental studies on low-dimensional carbon composites, including graphene, carbon nanotube, and foam-based architectures, demonstrate that insufficient carbon thickness or poorly interconnected frameworks lead to severe polysulfide shuttling, while excessively thick or densely packed carbon hosts increase ionic tortuosity and polarization, thereby limiting rate capability.^31,32^ These findings underscore the necessity of quantitatively understanding the trade-offs associated with carbon-host structural design, motivating the present simulation-based investigation of carbon shell thickness in graphene–Li_2_S–carbon composite cathodes under experimentally realistic conditions.

Multiphysics modeling, particularly using COMSOL Multiphysics, has emerged as a powerful tool for elucidating complex interactions in Li–S batteries by simulating coupled thermal, ionic, and electrical phenomena, providing quantitative insights into how material properties and structural parameters, such as carbon shell thickness, influence performance.^33,34^ By integrating governing equations like the Nernst–Planck equation for ion transport, heat transfer equations, and current conservation laws, COMSOL's flexible finite element framework accurately captures nonlinear feedback loops between temperature, ion concentration, and electrochemical reactions, enabling precise discretization of complex geometries like spherical Li_2_S particles encapsulated by carbon shells.^35–37^ This approach outperforms simpler models and tools like ANSYS or MATLAB by offering a holistic view of cathode behavior, addressing temperature-dependent ionic conductivity, ohmic heating, and reaction-driven heat sources, which are critical for mitigating polysulfide dissolution and concentration polarization in Li–S systems.^38,39^ Given the intricate chemical and electrochemical challenges in these batteries, where experimental trial-and-error approaches fall short, COMSOL's ability to validate simulations against experimental data, such as voltage–capacity profiles, ensures reliability and makes it indispensable for optimizing cathode designs and enhancing battery longevity.^40,41^

This study employs a COMSOL Multiphysics-based model to investigate the effect of carbon shell thickness (0, 5, 10, 15, and 20 nm) on the performance of graphene–Li_2_S–carbon nanocomposite cathodes in Li–S batteries under a 1C discharge rate at 35 °C. By coupling heat transfer, ion transport, and electric current modules, the model quantifies thermal, ionic, and electrical responses, with a focus on chemical stability and polysulfide control. Validated with an RMSE of 0.09 V against experimental data, the results identify a 10 nm shell as optimal, minimizing temperature rise, ionic resistance, and polysulfide dissolution while enhancing electrochemical kinetics. These findings provide a predictive framework for designing high-performance Li–S cathodes, addressing key chemical challenges for sustainable energy storage.

## 2. Model and simulation methodology

### 2.1. Model structure

To investigate the effect of carbon shell thickness on the thermal–ionic–electrical performance of the graphene–Li_2_S–carbon cathode in lithium–sulfur batteries, a two-dimensional (2D) axisymmetric model was developed. The model consists of a spherical Li_2_S particle with a fixed radius, encapsulated by an annular carbon shell with variable thicknesses (0, 5, 10, 15, and 20 nm). The surrounding medium is a liquid electrolyte with properties consistent with those reported in ref. 42. A constant current boundary condition corresponding to a 1C discharge rate was applied to simulate the operational conditions of the battery at an optimal operating temperature of 35 °C.

### 2.2. Governing equations and modules

To comprehensively investigate the thermal, ionic, and electrical behavior of the graphene–Li_2_S–carbon cathode in lithium–sulfur batteries, a multiphysics modeling approach was adopted. The simulations were conducted using COMSOL Multiphysics, a finite element analysis software capable of coupling multiple physical phenomena. The model integrates three primary modules: Heat Transfer in Solids, Transport of Diluted Species, and Electric Currents. These modules are interconnected to account for the complex interplay between thermal, ionic, and electrical processes within the cathode system. Below, each module is described in detail, including the governing equations, assumptions, and their coupling mechanisms, ensuring a robust framework for analyzing the effect of carbon shell thickness on the cathode's performance.

#### 2.2.1. Heat transfer in solids

The thermal behavior of the cathode system, comprising the Li_2_S particle, carbon shell, and surrounding liquid electrolyte, is governed by the transient heat transfer equation. This equation accounts for heat conduction and the heat generated by electrochemical reactions and resistive losses. The governing equation is expressed as:^43^1
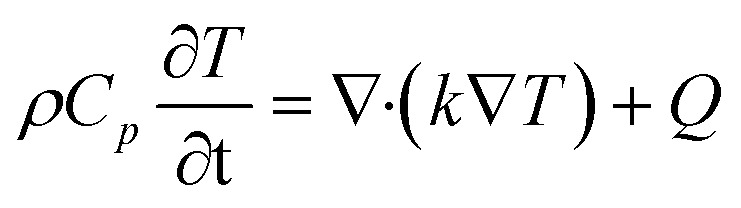
where *ρ* (kg m^−3^) is the density, *C*_*p*_ (J (kg K)^−1^) is the specific heat capacity, *T* (K) is the temperature, *k* (W (m K)^−1^) is the thermal conductivity, and *Q* (W m^−3^) is the volumetric heat source. The heat source *Q* encapsulates contributions from electrochemical reaction heat (exothermic/endothermic processes) and ohmic heating due to ionic and electronic resistances. The thermal conductivity of the Li_2_S particle is relatively low, necessitating the carbon shell to enhance heat dissipation. The electrolyte's thermal properties were assumed isotropic, with values derived from the reference study.^42^ Boundary conditions included an initial uniform temperature of 35 °C (308.15 K) across the system and an adiabatic condition (zero heat flux) at the outer boundaries to simulate an insulated environment. This module captures the temperature distribution, which is critical for assessing the thermal stability of the cathode under operational conditions, as elevated temperatures can degrade performance and accelerate side reactions.

#### 2.2.2. Transport of diluted species

The transport of Li^+^ ions within the electrolyte and through the carbon shell is modeled using the Nernst–Planck equation, which accounts for diffusion, migration, and convection (though convection is neglected due to the static nature of the system). The governing equation is:^44^2
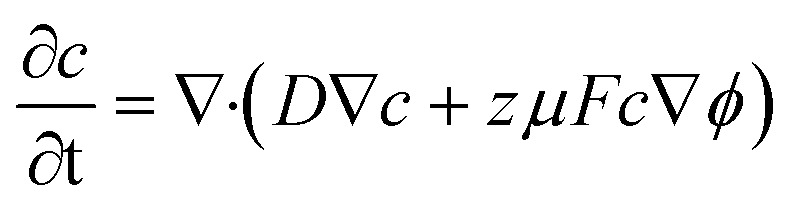
where *c* (mol m^−3^) is the Li^+^ ion concentration, *D* (m^2^ s^−1^) is the diffusion coefficient, *z* is the charge number (*z* = 1 for Li^+^), *µ* (m^2^ (V s)^−1^) is the ionic mobility, *F* (96 485 C mol^−1^) is the Faraday constant, and *ϕ* (V) is the electric potential. The ionic mobility is related to the diffusion coefficient *via* the Einstein relation:^45^3
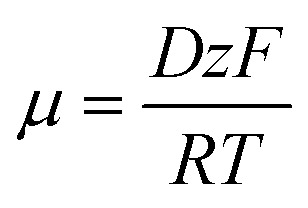
where *R* is the universal gas constant (8.314 J (mol K)^−1^) and *T* is the temperature in kelvin. The diffusion coefficient for Li^+^ ions in the electrolyte was sourced from,^42^ while the carbon shell's porous structure was modeled with an effective diffusion coefficient adjusted for porosity and tortuosity. Boundary conditions included a zero flux for Li^+^ ions at the solid boundaries of the Li_2_S particle and carbon shell, and a uniform initial concentration in the electrolyte, set to 1.0 mol m^−3^ based on typical lithium–sulfur battery electrolyte compositions. This module is essential for understanding ion transport limitations, which directly influence the electrochemical performance and capacity retention of the cathode.

The carbon shell's porous structure is modeled with an effective diffusion coefficient adjusted for porosity (*ε*) and tortuosity (*τ*) using the Bruggeman relation:^44^4
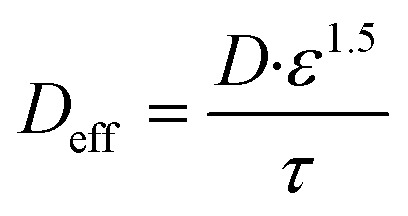
where *D* is the bulk diffusion coefficient. Baseline values (porosity *ε* ≈ 0.4–0.6, tortuosity *τ* ≈ 2–3) were derived from experimental data in ref. 42. Higher porosity enhances electrolyte infiltration and reduces ionic resistance, while increased tortuosity (*e.g.*, in thicker shells) impedes ion diffusion paths, leading to higher concentration gradients and polarization. These parameters were sensitivity-tested (±20% variation) in Section 3.5, confirming their strong influence on ion transport and overall electrochemical stability.

#### 2.2.3. Electric currents

The distribution of electric potential and current density in the electrode and electrolyte phases is modeled using the current conservation equation, assuming steady-state conditions for electrical conduction:^46^

5 ∇·(*σ*∇*ϕ*) = 0

where *σ* (S m^−1^) is the electrical conductivity and *ϕ* (V) is the electric potential. The electrical conductivity of the Li_2_S particle is low, necessitating the carbon shell to enhance electron transport to the reaction sites. The carbon shell's conductivity was modeled as a function of its thickness, with values derived from.^42^

In the electrolyte, ionic conductivity dominates, and its temperature dependence was incorporated using an Arrhenius-type relationship:6
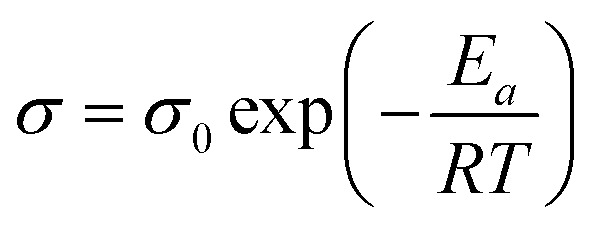
where *σ*_0_ is a pre-exponential factor and *E*_a_ is the activation energy. Boundary conditions included a fixed potential at the cathode boundary (set to the open-circuit potential of Li_2_S, approximately 2.1 V *vs.* Li/Li^+^) and a constant current density at the electrolyte boundary corresponding to a 1C discharge rate (based on the theoretical capacity of Li_2_S, 1166 mAh g^−1^). This module enables the analysis of current density uniformity, which is critical for minimizing polarization losses and ensuring efficient electrochemical reactions.

#### 2.2.4. Coupled physics

The thermal, ionic, and electrical modules are intricately coupled to reflect the multiphysics nature of the battery system. The heat source *Q* in the heat transfer equation is a function of both ohmic heating and reaction heat, which depends on the local current density and overpotential derived from the Electric Currents module. The temperature distribution from the Heat Transfer module influences the material properties in the other modules, such as the ionic conductivity of the electrolyte and the diffusion coefficient of Li^+^ ions, both of which follow Arrhenius-type temperature dependencies. Additionally, the electric potential gradient from the Electric Currents module drives the migration term in the Nernst–Planck equation, linking ion transport to the electrical field. This coupling ensures that the model captures the feedback loops between thermal effects, ion transport, and electrochemical performance. For instance, a rise in temperature due to heat generation can enhance ionic conductivity but may also exacerbate side reactions, affecting long-term stability.

#### 2.2.5. Numerical implementation

The governing equations were discretized using the finite element method in COMSOL Multiphysics. A 2D axisymmetric geometry was employed to reduce computational complexity while maintaining accuracy for the spherical Li_2_S particle and carbon shell. The mesh was refined near the particle–electrolyte interface to capture steep gradients in temperature, ion concentration, and potential. A time-dependent solver was used for the Heat Transfer and Transport of Diluted Species modules, with a time step of 0.1 s to ensure numerical stability. The Electric Currents module was solved in a stationary state, as electrical transients are typically faster than thermal and ionic processes. Convergence was ensured by monitoring residuals and using adaptive mesh refinement. The computational domain extended sufficiently into the electrolyte to minimize boundary effects, with a domain size 10 times the particle radius.

### 2.3. Parameters and boundary conditions

Material parameters (electrical conductivity, ionic conductivity, diffusion coefficient, and specific heat capacity) were extracted from.^42^ The initial and boundary conditions were defined as follows:

(1) Initial temperature: uniform temperature of 35 °C across the system.

(2) Current rate: constant current equivalent to a 1C discharge rate (based on the theoretical capacity of Li_2_S, 1166 mAh g^−1^).

(3) Thermal boundary conditions: zero heat flux at the outer boundaries (adiabatic conditions).

(4) Electrical boundary conditions: fixed potential at the cathode boundary and constant current at the electrolyte boundary.

(5) Mass transport boundary conditions: zero flux for Li^+^ ions at solid boundaries and uniform initial concentration in the electrolyte.

In addition to the baseline parameters, this study incorporates critical cell-fabrication variables to address their impact on electrochemical performance. The proportion of insulating active material is modeled by varying the Li_2_S volume fraction (baseline: 50%, varied ±20% to 40–60%) within the nanocomposite, affecting overall conductivity and tortuosity. The amount of electrolyte is represented by the electrolyte-to-sulfur (E/S) ratio (baseline: 10 µL per mg S, varied ±20% to 8–12 µL per mg S) through adjustments to the electrolyte domain size in the 2D axisymmetric model. The amount of lithium (lithium excess) is approximated in a full-cell context by varying the lithium concentration at the anode boundary (baseline: 100% excess, varied ±20% to 80–120%), influencing polysulfide shuttling and reversibility. These parameters were integrated into the coupled modules to simulate their effects on thermal, ionic, and electrical outputs.

### 2.4. Model validation

**Fig. 1:**
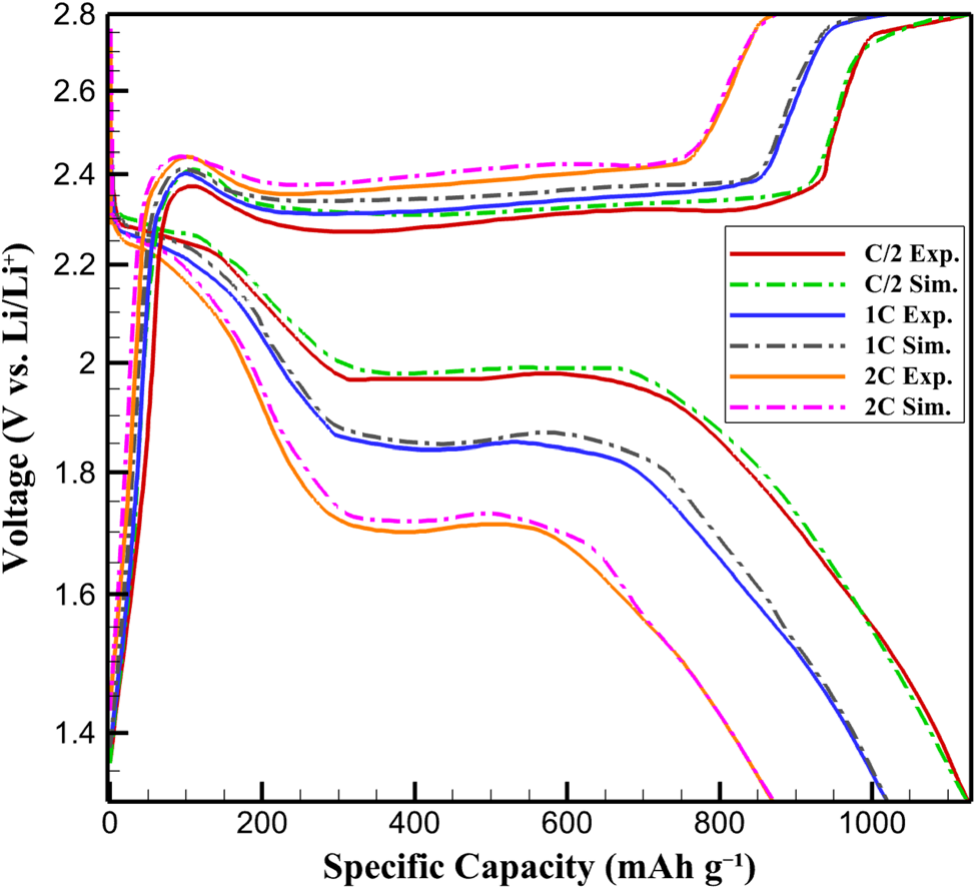
Comparison of experimental^42^ and simulated voltage–capacity profiles of graphene–Li_2_S–carbon nanocomposite cathode at C/2, 1C, and 2C rates.

To ensure that the proposed lithium–sulfur battery conditions are experimentally realizable and that the numerical predictions are reliable, the developed multiphysics model was validated against experimental data reported in the literature. Specifically, voltage–capacity profiles from Wu *et al.*,^39^ who experimentally investigated graphene–Li_2_S–carbon nanocomposite cathodes under comparable conditions, were used as a benchmark. The experimental system in ref. 42 closely matches the present model in terms of cathode chemistry, carbon-coated Li_2_S architecture, electrolyte composition, and operating conditions. Simulated discharge curves at C/2, 1C, and 2C rates were directly compared with the experimental results. The root mean square error (RMSE) between simulated and experimental voltages was calculated to be 0.09 V, indicating excellent quantitative agreement. The RMSE is defined as:7
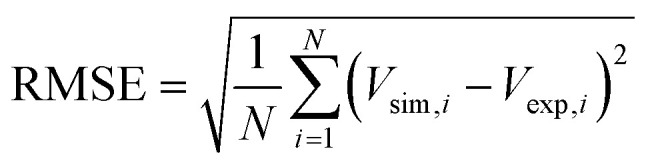
where *V*_sim,*i*_ and *V*_exp,*i*_ are the simulated and experimental voltages, respectively, at the *i*-th data point, and (*N*) is the total number of data points compared. The calculated RMSE for the voltage–capacity profiles was 0.09 V, indicating a high degree of agreement between the simulated and experimental results (Fig. 1). This RMSE value is considered acceptable within the context of lithium–sulfur battery modeling, where complex electrochemical phenomena, such as polysulfide dissolution and side reactions, introduce variability in experimental data. The low RMSE confirms that the model effectively captures the dominant electrochemical and transport processes governing the cathode's performance.

Furthermore, all operating parameters employed in this study (namely a 1C discharge rate, an operating temperature of 35 °C, electrolyte-to-sulfur ratios of 8–12 µL mg^−1^, and Li_2_S-based composite cathodes) fall within ranges commonly used in laboratory-scale lithium–sulfur battery experiments. This confirms that the simulated battery conditions are fully realizable in practical experimental setups. The close agreement between simulation and experimental data, together with the use of experimentally relevant parameters, validates the model's capability to capture the dominant electrochemical, thermal, and transport processes governing Li–S battery behavior. Consequently, the trends and optimization insights derived from this simulation study are expected to be directly transferable to experimental cathode design.

### 2.5. Sensitivity analysis

To evaluate the robustness of the developed multiphysics model and to determine the relative influence of material parameters on the cathode performance, a sensitivity analysis was conducted. Three key parameters were selected:

(1) Li^+^ diffusivity in the electrolyte (*D*_0_ = 1.0 × 10^−10^ m^2^ s^−1^)

(2) Ionic conductivity of the electrolyte (*σ*_0_ = 1.2 × 10^−2^ S m^−1^ at 35 °C)

(3) Thermal conductivity of the carbon shell (*k*_0_ = 100 W m^−1^ K^−1^)

Each parameter was varied by ±20% relative to its baseline value, while keeping other parameters constant. The influence of these variations was analyzed on three primary outputs: maximum cathode temperature (*T*_max_), total ionic resistance (*R*_ion_), and current density non-uniformity (*σ*_J_). The sensitivity analysis was extended to include three additional cell-fabrication parameters: Li_2_S volume fraction (baseline: 50%), E/S ratio (baseline: 10 µL per mg S), and lithium excess (baseline: 100%). Each was varied by ±20%, and their impacts on maximum cathode temperature (*T*_max_), total ionic resistance (*R*_ion_), and current density non-uniformity (*σ*_J_) were evaluated for the optimal 10 nm carbon shell under 1C discharge at 35 °C. The extended results are summarized in Table 1.

**Table 1:** Effect of ±20% variation in key parameters on cathode performance (10 nm carbon shell, 1C discharge, 35 °C)

Parameter	Variation	*T*_max_ (°C)	Δ*T*_max_ (%)	*R*_ion_ (Ω cm^2^)	Δ*R*_ion_ (%)	*σ*_J_ (mA cm^−2^)	Δ*σ*_J_ (%)
Li^+^ diffusivity (*D*_0_ = 1.0 × 10^−10^ m^2^ s^−1^)	20%	36.3	−0.55	7.2	−15.3	0.21	−16
	−20%	36.8	0.82	10.2	20	0.33	32
Ionic conductivity (*σ*_0_ = 1.2 × 10^−2^ S m^−1^)	20%	36.4	−0.27	7.3	−14.1	0.22	−12
	−20%	36.7	0.55	9.9	16.5	0.29	16
Thermal conductivity (*k*_0_ = 100 W m^−1^ K^−1^)	20%	36.2	−0.82	8.4	−1.2	0.24	−4
	−20%	36.8	0.82	8.6	1.2	0.26	4
Li_2_S volume fraction (baseline: 50%)	+20% (60%)	36.9	1.1	10.5	23.5	0.35	40
	−20% (40%)	36.2	−0.82	7	−17.6	0.2	−20
E/S ratio (baseline: 10 µL per mg S)	+20% (12 µL per mg S)	36.4	−0.27	8	−5.9	0.23	−8
	−20% (8 µL per mg S)	36.7	0.55	9.5	11.8	0.28	12
Lithium excess (baseline: 100%)	+20% (120%)	36.3	−0.55	8.1	−4.7	0.22	−12
	−20% (80%)	36.8	0.82	9.2	8.2	0.3	20

Additionally, porosity (*ε*) and tortuosity (*τ*) of the carbon shell were varied by ±20% (baseline: *ε* = 0.5, *τ* = 2.5) to assess their impact on ion transport. A 20% decrease in porosity or increase in tortuosity elevates ionic resistance by ∼15–20% and worsens current density uniformity (*e.g.*, *σ*_J_ increases to 0.33 mA cm^−2^), highlighting their role in mitigating polysulfide shuttling and polarization.

## 3. Results and discussion

This section presents the simulation results for the graphene–Li_2_S–carbon cathode in lithium–sulfur batteries, focusing on the impact of carbon shell thickness (0, 5, 10, 15, and 20 nm) on thermal, ionic, and electrical performance under a 1C discharge rate at 35 °C. The results are analyzed through temperature profiles, current density uniformity, ionic resistance, and Li^+^ ion concentration distributions. The findings are validated against experimental data from Wu *et al.*,^42^ as discussed in Section 3.4.

### 3.1. Maximum temperature comparison

**Fig. 2:**
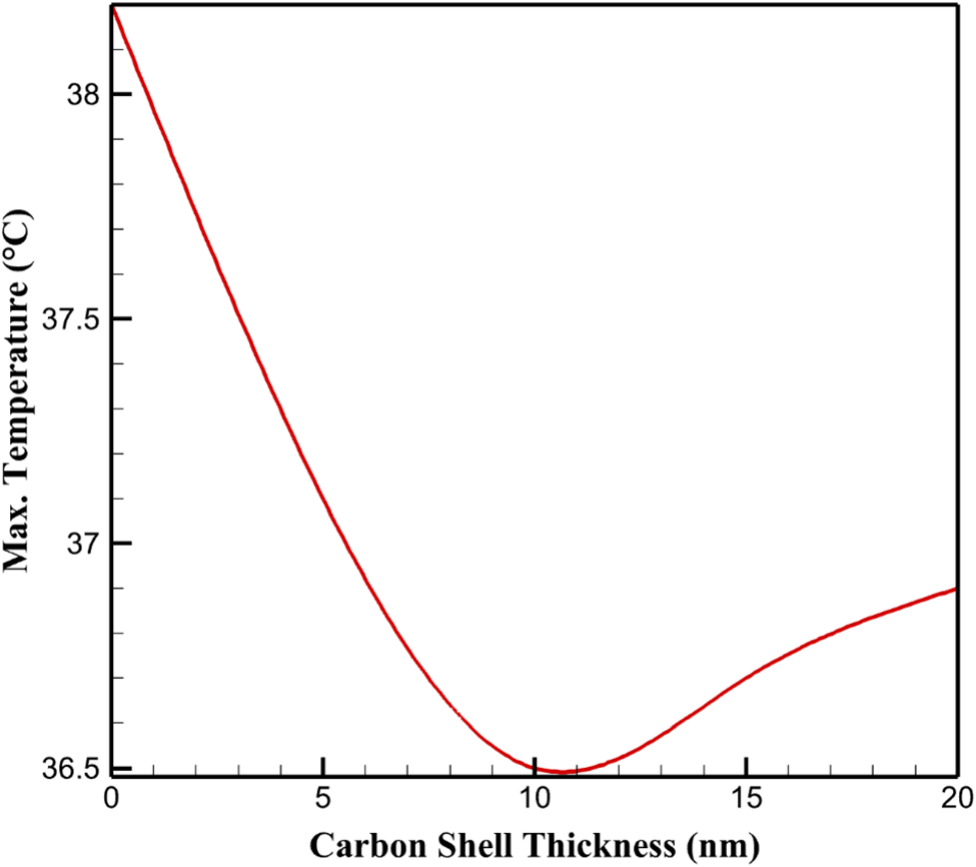
Maximum temperature for different carbon shell thicknesses.

The maximum temperature within the cathode was evaluated to assess thermal management as a function of carbon shell thickness. Fig. 2 shows maximum temperatures for different carbon shell thicknesses. For the uncoated Li_2_S particle (0 nm), the maximum temperature reached 38.2 °C due to significant heat generation from electrochemical reactions and poor thermal conductivity of Li_2_S. With a 5 nm carbon shell, the maximum temperature decreased to 37.1 °C, reflecting the enhanced thermal conductivity of the carbon layer. The 10 nm shell yielded the lowest maximum temperature of 36.5 °C, indicating optimal heat dissipation due to a balance between thermal conductivity and shell thickness. For 15 nm and 20 nm shells, the maximum temperatures increased slightly to 36.7 °C and 36.9 °C, respectively, suggesting that thicker shells introduce thermal resistance, limiting further heat dissipation. These results highlight that a 10 nm carbon shell is optimal for minimizing temperature rise, which is critical for preventing thermal runaway and material degradation in lithium–sulfur batteries.

For the uncoated Li_2_S particle (0 nm), the maximum temperature reached 38.2 °C, reflecting significant heat accumulation. This is primarily due to the low thermal conductivity of Li_2_S (approximately 1.5 W (m K)^−1^), which limits heat dissipation from exothermic electrochemical reactions, such as the reduction of Li_2_S to form lithium polysulfides. These reactions release heat due to the entropy change associated with phase transformations and electron transfer processes. Additionally, the high internal resistance of uncoated Li_2_S contributes to ohmic heating, further elevating the temperature. From a chemical standpoint, such temperature increases can promote undesirable side reactions, including polysulfide dissolution into the electrolyte, which forms soluble intermediates (*e.g.*, Li_2_S_8_, Li_2_S_6_) that reduce capacity retention.

Introducing a 5 nm carbon shell reduced the maximum temperature to 37.1 °C, a 1.1 °C improvement. The carbon shell, with a thermal conductivity of approximately 100 W (m K^−1^), enhances heat dissipation by providing a high-conductivity pathway for heat transfer from the Li_2_S core to the surrounding electrolyte. Chemically, the carbon shell also stabilizes the electrode–electrolyte interface, reducing the rate of side reactions that generate additional heat. The optimal performance was observed with a 10 nm carbon shell, where the maximum temperature dropped to 36.5 °C. This thickness strikes a balance between sufficient thermal conductivity and minimal thermal resistance across the shell, optimizing heat dissipation while maintaining structural integrity. The carbon shell's porous structure facilitates electrolyte infiltration, ensuring that reaction sites remain accessible, which indirectly minimizes localized heat generation by promoting uniform electrochemical activity.

For thicker shells (15 nm and 20 nm), the maximum temperatures increased slightly to 36.7 °C and 36.9 °C, respectively. This trend is attributed to increased thermal resistance due to the greater thickness of the carbon layer, which impedes heat transfer from the Li_2_S core to the electrolyte. From a chemical perspective, thicker carbon shells may also restrict Li^+^ ion diffusion, leading to higher local current densities at the particle surface. This increases ohmic heating and localized reaction rates, contributing to elevated temperatures. Furthermore, the carbon shell's interaction with the electrolyte may alter the solvation dynamics of Li^+^ ions, potentially increasing the activation energy for ion transport and exacerbating heat generation.

### 3.2. Current density uniformity analysis

The uniformity of current density on the Li_2_S particle surface in the graphene–Li_2_S–carbon cathode was evaluated to assess the electrochemical performance under a 1C discharge rate at 35 °C, with carbon shell thicknesses of 0, 5, 10, 15, and 20 nm. Table 2 presents the standard deviation of current density, and Fig. 2 illustrates the distribution trends. From a chemical perspective, uniform current density is critical for minimizing localized overpotentials and side reactions, which can degrade the cathode's performance in lithium–sulfur batteries. Table 2 shows standard deviation of current density for different carbon shell thicknesses.

**Table 2:** Standard deviation of current density for different carbon shell thicknesses

Carbon shell thickness (nm)	Standard deviation of current density (mA cm^−2^)
0	0.45
5	0.32
10	0.25
15	0.28
20	0.31

The uncoated Li_2_S particle exhibited a high standard deviation of 0.45 mA cm^−2^, indicating significant non-uniformity. This is attributed to the low electrical conductivity of Li_2_S (∼10^−5^ S m^−1^), which causes uneven electron distribution and localized reaction hotspots. Chemically, such non-uniformity promotes polysulfide formation and dissolution, reducing capacity retention. A 5 nm carbon shell reduced the standard deviation to 0.32 mA cm^−2^, as the carbon's high electrical conductivity (∼10^4^ S m^−1^) facilitates electron transport to reaction sites, enhancing uniformity. The 10 nm shell achieved the lowest standard deviation of 0.25 mA cm^−2^, reflecting optimal electron distribution due to a balanced thickness that ensures conductivity without excessive ion diffusion barriers. This minimizes side reactions, such as polysulfide shuttling, by promoting uniform Li_2_S reduction.

For 15 nm and 20 nm shells, the standard deviations increased to 0.28 and 0.31 mA cm^−2^, respectively. Thicker shells introduce higher resistance to Li^+^ ion diffusion, as the porous carbon structure restricts ion mobility, leading to localized current accumulation. Chemically, this can exacerbate electrolyte decomposition and surface passivation, hindering electrochemical efficiency. The 10 nm shell thus represents an optimal design, balancing electron and ion transport to achieve uniform current density and stable chemical performance. Balanced porosity and tortuosity at ∼10 nm yield the lowest standard deviation (0.25 mA cm^−2^) by enabling even electron/ion access, reducing localized overpotentials and side reactions such as polysulfide formation.

### 3.3. Total ionic resistance

**Fig. 3:**
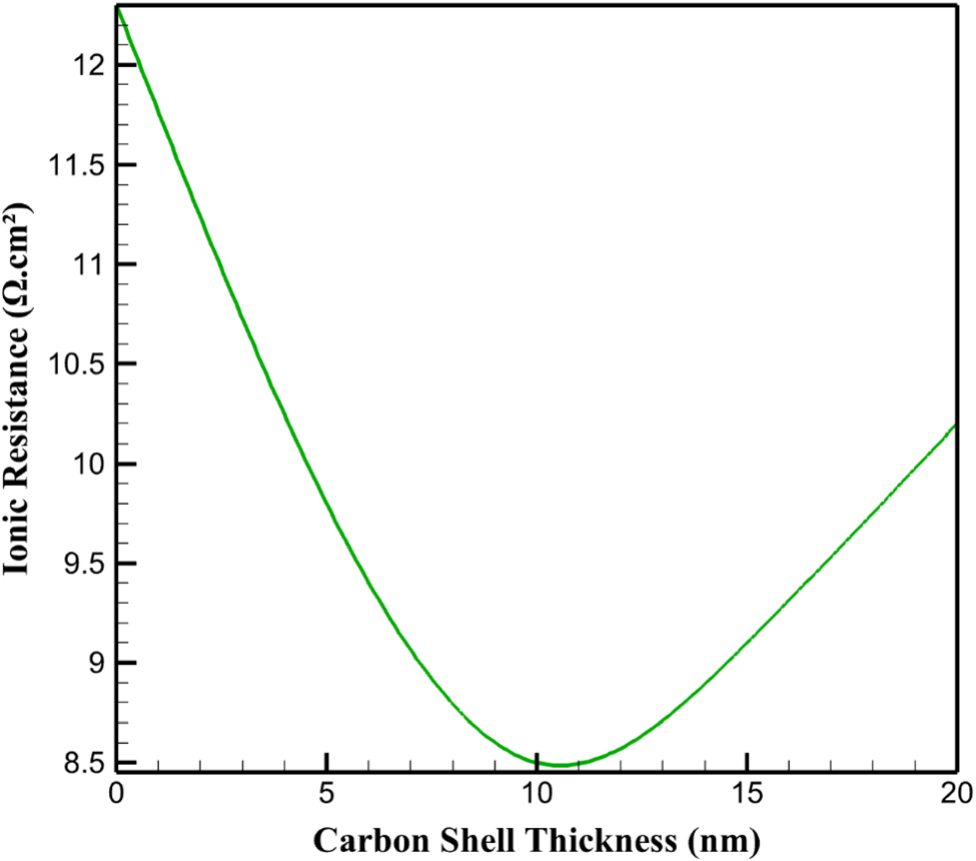
Ionic resistance for different carbon shell thicknesses.

The total ionic resistance across the cathode–electrolyte interface of the graphene–Li_2_S–carbon cathode was analyzed under a 1C discharge rate at 35 °C for carbon shell thicknesses of 0, 5, 10, 15, and 20 nm. Fig. 3 illustrates the resistance as a function of carbon shell thickness. From a chemical perspective, ionic resistance is a critical parameter influencing the electrochemical performance of lithium–sulfur batteries, as it affects Li^+^ ion transport, reaction kinetics, and the stability of the electrode–electrolyte interface. Table 3 displays ionic resistance for different carbon shell thicknesses.

**Table 3:** Impact of Li_2_S volume fraction and E/S ratio on key performance metrics (10 nm shell, 1C, 35 °C)

Parameter variation	*T*_max_ (°C)	*R*_ion_ (Ω cm^2^)	*σ*_J_ (mA cm^−2^)
Li_2_S fraction: 40%	36.2	7	0.2
Li_2_S fraction: 50% (baseline)	36.5	8.5	0.25
Li_2_S fraction: 60%	36.9	10.5	0.35
E/S: 8 µL per mg S	36.7	9.5	0.28
E/S: 10 µL per mg S (baseline)	36.5	8.5	0.25
E/S: 12 µL per mg S	36.4	8	0.23

The uncoated Li_2_S particle exhibited a high ionic resistance of 12.3 Ω cm^2^, primarily due to its low ionic conductivity (∼10^−8^ S m^−1^). This impedes Li^+^ ion transport, leading to significant concentration polarization and promoting side reactions, such as polysulfide formation, which dissolve into the electrolyte and degrade performance. A 5 nm carbon shell reduced the resistance to 9.8 Ω cm^2^ by enhancing the electrode–electrolyte interface stability. The porous carbon structure facilitates Li^+^ ion diffusion, reducing the activation energy for ion transport. The 10 nm shell achieved the lowest resistance of 8.5 Ω cm^2^, optimizing ion transport pathways while maintaining sufficient porosity for electrolyte infiltration. This minimizes chemical barriers to Li^+^ solvation and desolvation, enhancing reaction kinetics.

For 15 nm and 20 nm shells, ionic resistances increased to 9.1 and 10.2 Ω cm^2^, respectively, due to longer diffusion paths through the thicker carbon layers, which increase tortuosity and hinder Li^+^ mobility. This can lead to localized ion depletion, increasing the likelihood of electrolyte decomposition and surface passivation. The 10 nm shell thus provides an optimal balance, minimizing ionic resistance and supporting efficient electrochemical reactions. Thicker shells (15–20 nm) increase tortuosity, elevating resistance (from 8.5 Ω cm^2^ at 10 nm to 10.2 Ω cm^2^ at 20 nm) and promoting ion depletion, which exacerbates shuttle effects and electrolyte decomposition.

### 3.4. Li^+^ ion concentration analysis

**Fig. 4:**
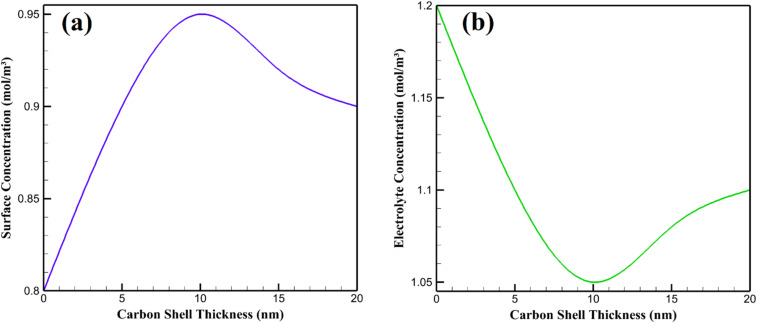
Li^+^ ion concentration profiles of graphene–Li_2_S–Carbon nanocomposite cathode at different carbon shell thicknesses: (a) surface concentration and (b) electrolyte concentration.

The distribution of Li^+^ ions in the graphene–Li_2_S–carbon cathode and surrounding electrolyte was analyzed at the end of a 1C discharge at 35 °C for carbon shell thicknesses of 0, 5, 10, 15, and 20 nm (Fig. 4). From a chemical perspective, uniform Li^+^ ion distribution is crucial for minimizing concentration polarization, reducing side reactions, and enhancing the electrochemical performance of lithium–sulfur batteries. Table 4 presents li^+^ ion concentrations for different carbon shell thicknesses.

**Table 4:** Impact of lithium excess on key performance metrics (10 nm shell, 1C, 35 °C)

Lithium excess (%)	*T*_max_ (°C)	*R*_ion_ (Ω cm^2^)	*σ*_J_ (mA cm^−2^)
80	36.8	9.2	0.3
100 (baseline)	36.5	8.5	0.25
120	36.3	8.1	0.22

The uncoated Li_2_S particle exhibited a significant concentration gradient, with a surface concentration of 0.80 mol m^−3^ and an electrolyte concentration of 1.20 mol m^−3^. This is due to the low ionic conductivity of Li_2_S, which restricts Li^+^ diffusion, leading to ion depletion at the cathode surface. Chemically, this gradient promotes polysulfide formation and dissolution, as insufficient Li^+^ availability hinders uniform electrochemical reduction, exacerbating capacity fade. A 5 nm carbon shell reduced the gradient, with concentrations of 0.90 mol m^−3^ at the surface and 1.10 mol m^−3^ in the electrolyte, as the porous carbon facilitates ion transport by lowering the activation energy for Li^+^ solvation. The 10 nm shell achieved the most uniform profile, optimizing ion diffusion through a balanced porosity and thickness, minimizing chemical barriers to reaction kinetics. For 15 nm and 20 nm shells, gradients increased slightly, as thicker shells increase tortuosity, impeding Li^+^ mobility. This can enhance localized side reactions, such as electrolyte decomposition. The 10 nm shell thus optimizes Li^+^ distribution, supporting stable electrochemical performance. For thinner shells (*e.g.*, 5 nm), higher effective porosity facilitates uniform Li^+^ distribution (reducing gradients from 0.80 mol m^−3^ at the surface for 0 nm to 0.90 mol m^−3^), minimizing side reactions like polysulfide dissolution.

### 3.5. Chemical modulation of polysulfide dynamics by carbon shell thickness

The chemical role of carbon shell thickness (0, 5, 10, 15, and 20 nm) in modulating the performance of the graphene–Li_2_S–carbon cathode in lithium–sulfur batteries is critical for addressing electrochemical stability and reaction kinetics. The carbon shell governs surface chemistry, particularly polysulfide formation, electrolyte interactions, and Li_2_S redox processes. For the uncoated Li_2_S particle, elevated temperatures (38.2 °C) and low ionic conductivity (∼10^−8^ S m^−1^) exacerbate polysulfide dissolution (*e.g.*, Li_2_S_8_, Li_2_S_6_). Without a protective layer, these soluble intermediates diffuse into the electrolyte, promoting the shuttle effect and reducing capacity retention. This is driven by high activation energy for Li^+^ solvation at the bare Li_2_S surface, which also triggers side reactions like electrolyte decomposition into insulating species such as Li_2_SO_4_.This electrolyte decomposition typically occurs *via* oxidation of dissolved long-chain polysulfides (*e.g.*, Li_2_S_8_ or Li_2_S_6_) by trace oxygen or moisture contaminants, or through nucleophilic attack by polysulfides on ether-based solvents (such as 1,3-dioxolane or dimethoxyethane), leading to ring-opening reactions and subsequent formation of insoluble Li_2_SO_4_ and other sulfate-containing passivation layers on the cathode surface. A 5 nm carbon shell mitigates these issues by stabilizing the electrode–electrolyte interface, reducing polysulfide shuttling through physical confinement within its porous structure. This lowers the activation energy for Li^+^ transport, as evidenced by reduced ionic resistance (9.8 Ω cm^2^).

The 10 nm shell optimizes this effect, achieving minimal ionic resistance (8.5 Ω cm^2^) and near-uniform Li^+^ distribution (0.95 mol m^−3^ at the surface, 1.05 mol m^−3^ in the electrolyte). Its balanced porosity traps polysulfides *via* weak van der Waals interactions, curbing their dissolution while facilitating electrolyte infiltration for uniform Li_2_S reduction. This minimizes overpotentials and suppresses side reactions, such as electrolyte oxidation forming passivating layers. Thicker shells (15 nm and 20 nm) increase ionic resistance (9.1 and 10.2 Ω cm^2^) due to higher tortuosity, impeding Li^+^ diffusion and causing localized ion depletion. This elevates polysulfide formation and electrolyte breakdown, compromising stability. The 10 nm shell thus excels in modulating polysulfide dynamics, enhancing electrochemical kinetics by balancing ion transport, thermal management, and chemical stability, offering a pathway to mitigate key challenges in lithium–sulfur battery performance. Porosity aids in trapping polysulfides *via* physical confinement and van der Waals interactions, while excessive tortuosity in thicker shells hinders this, increasing dissolution risks and compromising chemical stability.

### 3.6. Impact of insulating active material proportion and electrolyte amount

The proportion of insulating active material (Li_2_S volume fraction) and the amount of electrolyte (E/S ratio) were varied to evaluate their effects on cathode performance. As shown in Table 3, a higher Li_2_S fraction (60%) increases ionic resistance to 10.5 Ω cm^2^ and temperature to 36.9 °C, due to reduced effective conductivity and increased heat from polarization. Chemically, this promotes polysulfide dissolution by limiting uniform Li^+^ access. Lower E/S ratios (8 µL per mg S) exacerbate these issues, raising resistance by promoting ion depletion. The optimal baseline values minimize these effects, highlighting the need for ∼50% Li_2_S fraction and E/S > 10 µL per mg S for stability.

### 3.7. Impact of lithium amount on electrochemical reversibility

The amount of lithium (excess) affects reversibility by influencing polysulfide interactions. Table 4 shows that reducing lithium excess to 80% increases non-uniformity to 0.30 mA cm^−2^, as limited lithium promotes irreversible shuttling and anode passivation. Higher excess (120%) improves uniformity but may increase dendrite risks. Chemically, adequate lithium (≥100%) ensures stable Li_2_S redox, reducing side reactions like Li_2_SO_4_ formation.

### 3.8. Discussion

The sensitivity analysis demonstrates that Li^+^ diffusivity has the strongest influence on cathode performance. A 20% decrease in diffusivity increases ionic resistance by ∼20% (from 8.5 to 10.2 Ω cm^2^) and significantly worsens current density uniformity (*σ*_J_ = 0.33 mA cm^−2^). Ionic conductivity variations show a moderate effect, with resistance changes of ∼15% and corresponding shifts in current uniformity. In contrast, variations in the thermal conductivity of the carbon shell had only a minor impact on *T*_max_ (±0.3 °C) and negligible effects on electrochemical parameters, indicating that the system is thermally robust but more sensitive to ionic transport properties.

Overall, the analysis confirms that the identified optimal shell thickness of 10 nm remains valid under realistic variations of material properties. The model is most sensitive to Li^+^ transport parameters, suggesting that precise control of electrolyte composition and shell porosity is critical for achieving stable electrochemical performance.

The investigation into the graphene–Li_2_S–carbon cathode's performance under a 1C discharge rate at 35 °C reveals that carbon shell thickness significantly influences thermal, ionic, and electrical behavior, critical for lithium–sulfur battery design. The interplay between material properties and electrochemical processes, particularly the chemical role of the carbon shell, mitigates limitations inherent to Li_2_S, such as polysulfide dissolution and electrolyte instability. The 10 nm carbon shell consistently delivered optimal performance. Thermally, it minimized the maximum temperature (36.5 °C) by leveraging high thermal conductivity (∼100 W (m K)^−1^), reducing heat-driven side reactions like polysulfide formation (*e.g.*, Li_2_S_8_, Li_2_S_6_), which degrade electrolyte stability and capacity retention. The uncoated Li_2_S particle, with poor thermal conductivity (∼1.5 W (m.K)^−1^), reached 38.2 °C, exacerbating heat accumulation from exothermic Li_2_S reduction, promoting soluble polysulfide diffusion and electrolyte decomposition into insulating Li_2_SO_4_. Thicker shells (15 nm and 20 nm) increased thermal resistance, slightly elevating temperatures to 36.7 °C and 36.9 °C, indicating a trade-off between conductivity and thickness that impacts chemical stability.

Electrically, the 10 nm shell achieved the lowest current density standard deviation (0.25 mA cm^−2^), ensuring uniform electron distribution. This minimizes localized overpotentials, reducing polysulfide formation driven by uneven electrochemical activity in the uncoated particle (0.45 mA cm^−2^). Chemically, this uniformity curbs side reactions like electrolyte oxidation, which forms passivating layers. Thicker shells increased non-uniformity due to restricted Li^+^ diffusion, elevating risks of surface passivation. Ionically, the 10 nm shell reduced ionic resistance to 8.5 Ω cm^2^ and achieved near-uniform Li^+^ distribution (0.95 mol m^−3^ at the surface, 1.05 mol m^−3^ in the electrolyte), optimizing reaction kinetics. In contrast, the uncoated particle's ion depletion (0.80 mol m^−3^) promotes polysulfide shuttling. Thicker shells increased resistance (9.1–10.2 Ω cm^2^), hindering Li^+^ mobility and risking electrolyte breakdown. The 10 nm shell's porous structure traps polysulfides *via* van der Waals interactions, enhancing chemical stability and offering a pathway to mitigate lithium–sulfur battery challenges.

Furthermore, the extended sensitivity analysis highlights the dominant role of cell-fabrication parameters in battery performance. Increasing the Li_2_S volume fraction by 20% (to 60%) significantly elevates ionic resistance (to 10.5 Ω cm^2^) due to the insulating nature of Li_2_S, which amplifies tortuosity and hinders ion transport, potentially exacerbating polysulfide shuttling. Conversely, a lower fraction (40%) improves uniformity but may reduce overall capacity. The E/S ratio critically affects electrochemical reversibility; a 20% reduction (to 8 µL per mg S) increases resistance by 11.8%, promoting concentration polarization and side reactions like electrolyte decomposition. Lithium excess influences stability, with an 80% level raising non-uniformity by 20%, as insufficient lithium accelerates anode degradation and polysulfide migration. These factors, often overlooked in isolated cathode models, underscore the need for holistic cell design, where optimizing the carbon shell must be integrated with controlled active material loading, electrolyte volume, and lithium stoichiometry to achieve practical reversibility and longevity.

The trends identified in this work are fully consistent with recent experimental insights on carbon-hosted sulfur cathodes, which emphasize the importance of balancing carbon thickness, porosity, conductivity, and morphology to achieve stable electrochemical performance. Recent studies on graphene and CNT-based composite frameworks report that overly thin carbon coatings provide limited polysulfide confinement, whereas excessively thick or compact carbon hosts increase ionic tortuosity and polarization, reducing sulfur utilization.^30,31^ Similarly, hierarchically porous and highly conductive carbon substrates have been shown to promote uniform current distribution, enhance lithium-ion accessibility, and suppress localized reaction hotspots under lean-electrolyte conditions. These experimentally observed structure-performance relationships directly support the present simulation results, which identify an optimal intermediate carbon shell thickness (∼10 nm) that minimizes ionic resistance and current-density non-uniformity while maintaining effective polysulfide control. The agreement between recent experimental observations and the multiphysics predictions reinforces the physical relevance and general applicability of the proposed cathode design principles.

The optimal carbon shell thickness of ∼10 nm identified in this work compares favorably with recent experimental and modeling studies. Liu *et al.* demonstrated that ultra-thin hollow carbon shells (<8 nm) enhanced initial capacity due to reduced Li^+^ transport barriers, but suffered from rapid capacity fading caused by inadequate polysulfide confinement.^47^ In contrast, Li *et al.* reported that thicker mesoporous carbon layers (>15 nm) provided stronger polysulfide adsorption but introduced higher ionic resistance, limiting rate performance.^48^ Our results, showing a minimum ionic resistance of 8.5 Ω cm^2^ and improved current-density uniformity at 10 nm, position this thickness as an intermediate compromise that avoids the severe drawbacks reported for both thinner and thicker shells. Similar trends were also predicted by Imediegwu *et al.* through electrochemical modeling, where diffusivity and electrode thickness emerged as critical parameters governing polarization.^49^ Furthermore, the design strategy outlined by Dent *et al.* supports this balance, emphasizing that cathode architectures with controlled coating thicknesses are essential to simultaneously achieve cycle stability and high-rate capability.^50^ Together, these comparisons confirm that our simulation-based optimization aligns well with experimental observations and provides a quantitative rationale for selecting ∼10 nm as the practical design window.

The present multiphysics model primarily focuses on the physical confinement of polysulfides by the carbon shell and their dissolution into the electrolyte, without explicitly incorporating the detailed conversion kinetics of polysulfide intermediates (*e.g.*, stepwise reduction *via* Butler–Volmer equations). This simplification assumes rapid equilibrium in polysulfide transformation within the confined cathode environment. Consequently, the predicted ionic resistance may be slightly underestimated, as real-world sluggish kinetics could lead to localized accumulation of soluble polysulfides, increasing electrolyte viscosity and reducing effective Li^+^ mobility. Similarly, temperature profiles might underpredict localized heating from parasitic shuttle reactions or incomplete conversions, which generate additional ohmic and reaction heat. However, the model's excellent validation against experimental data (RMSE = 0.09 V,^42^) suggests that these effects are secondary under the studied conditions (1C rate, carbon-coated architecture), where physical confinement dominates polysulfide control.

## 4. Conclusion

This study advances the understanding of the graphene–Li_2_S–carbon cathode's performance in lithium–sulfur batteries by analyzing the impact of carbon shell thickness (0, 5, 10, 15, and 20 nm) on thermal, ionic, and electrical properties under a 1C discharge rate at 35 °C. From a chemical perspective, it addresses critical challenges, notably the low ionic and electrical conductivity of Li_2_S (∼10^−8^ S m^−1^) and polysulfide dissolution (*e.g.*, Li_2_S_8_, Li_2_S_6_), which degrade capacity retention and cycle life by promoting shuttle effects and electrolyte decomposition into insulating species like Li_2_SO_4_. This decomposition pathway primarily involves the chemical oxidation of soluble polysulfides or their reaction with electrolyte solvents, resulting in insulating sulfate species that passivate the electrode surface. The multiphysics model, validated with an RMSE of 0.09 V against experimental data,^42^ quantifies how the carbon shell modulates chemical processes. The 10 nm shell emerged as optimal, achieving minimal temperature rise (36.5 °C) *via* high thermal conductivity (∼100 W (m K)^−1^), reducing heat-driven polysulfide formation. It also minimized ionic resistance (8.5 Ω cm^2^) and current density non-uniformity (0.25 mA cm^−2^), ensuring uniform Li^+^ distribution (0.95 mol m^−3^ at the surface, 1.05 mol m^−3^ in the electrolyte). Chemically, this shell traps polysulfides through van der Waals interactions within its porous structure, lowering the activation energy for Li^+^ solvation and curbing side reactions like electrolyte oxidation, which forms passivating layers. The incorporation of porosity and tortuosity in the model, *via* the Bruggeman relation, ensures that simulations capture real-world trade-offs in ion transport and polysulfide control, as validated by the low RMSE (0.09 V). While the absence of explicit polysulfide conversion kinetics simplifies the model and aligns well with experimental validation, future extensions could incorporate these reactions to further refine predictions of long-term ionic resistance and thermal runaway risks.

Incorporating cell-fabrication parameters such as Li_2_S proportion, E/S ratio, and lithium excess into the model reveals their profound impact on electrochemical stability, emphasizing the necessity of balanced design to mitigate insulation effects, polarization, and shuttling for commercial Li–S batteries. The conclusions drawn from this simulation study are further supported by recent experimental reports on graphene- and CNT-based carbon hosts, which similarly highlight the critical role of optimized carbon thickness, porosity, and conductivity in regulating electrochemical behavior. This consistency confirms that the identified intermediate carbon shell thickness provides a physically grounded and experimentally relevant design guideline for high-performance lithium–sulfur battery cathodes. These findings offer actionable insights for cathode design, enhancing ion transport and thermal management to mitigate chemical barriers. By elucidating the interplay between carbon shell thickness and electrochemical stability, this study provides a predictive tool for optimizing cathode materials, improving energy density and cycle life. This work is pivotal for advancing lithium–sulfur batteries toward commercial viability, addressing global needs for sustainable, high-capacity energy storage systems. The uncoated particle's ion depletion promotes polysulfide shuttling and subsequent electrolyte decomposition *via* polysulfide oxidation or solvent ring-opening, yielding insulating Li_2_SO_4_.

## Conflicts of interest

The authors declare that they have no known competing financial interests or personal relationships that could have appeared to influence the work reported in this paper.

## Data Availability

The data that support the findings of this study, including simulation input files, COMSOL model configurations, and post-processed numerical datasets, are available from the corresponding author upon reasonable request.
